# Biomimetic supercontainers for size-selective electrochemical sensing of molecular ions

**DOI:** 10.1038/srep45786

**Published:** 2017-04-10

**Authors:** Nathan L. Netzer, Indrek Must, Yupu Qiao, Shi-Li Zhang, Zhenqiang Wang, Zhen Zhang

**Affiliations:** 1 Solid-State Electronics, The Ångström Laboratory, Uppsala University, SE-751 21, Uppsala, Sweden; 2 Department of Chemistry, The University of South Dakota, 414 E. Clark St., Vermillion, SD 57069, United States

## Abstract

New ionophores are essential for advancing the art of selective ion sensing. Metal-organic supercontainers (MOSCs), a new family of biomimetic coordination capsules designed using sulfonylcalix[4]arenes as container precursors, are known for their tunable molecular recognition capabilities towards an array of guests. Herein, we demonstrate the use of MOSCs as a new class of size-selective ionophores dedicated to electrochemical sensing of molecular ions. Specifically, a MOSC molecule with its cavities matching the size of methylene blue (MB^+^), a versatile organic molecule used for bio-recognition, was incorporated into a polymeric mixed-matrix membrane and used as an ion-selective electrode. This MOSC-incorporated electrode showed a near-Nernstian potentiometric response to MB^+^ in the nano- to micro-molar range. The exceptional size-selectivity was also evident through contrast studies. To demonstrate the practical utility of our approach, a simulated wastewater experiment was conducted using water from the Fyris River (Sweden). It not only showed a near-Nernstian response to MB^+^ but also revealed a possible method for potentiometric titration of the redox indicator. Our study thus represents a new paradigm for the rational design of ionophores that can rapidly and precisely monitor molecular ions relevant to environmental, biomedical, and other related areas.

Container molecules with well-defined nanocavities that are capable of hosting one or more guest molecules have attracted immense interest in recent years due to their exceptional performance in a wide variety of application areas such as gas storage and separation^1^,^2^,^3^,^4^, catalysis^5^,^6^,^7^,^8^, and sensing^8^,^9^,^10^. Inspired by the unique structure of spherical viruses that feature an *endo* (internal) cavity for storage of genetic materials and several *exo* (external) cavities for the recognition of target guests, we previously designed a new family of coordination container molecules termed metal-organic supercontainers (MOSCs)^11^,^12^,^13^,^14^,^15^. Thus far, four prototypes of MOSCs have been synthesized, which feature structurally similar multi-pore architecture to spherical viruses by comprising both *endo*- and *exo*-cavities. The presence of such hierarchical cavities as well as their chemical tunability equips these molecules with the opportunity to function as extremely efficient biomimetic host systems for molecular guests.

Concurrently, supramolecular host materials have become increasingly interesting for the electrochemical ion sensing field due to their favorable binding sites^16^,^17^. In particular, apart from possessing favorable binding sites with chemical tunability, MOSCs also express solution processability while preserving their unique combination of *endo*- and *exo*-cavities. Thus, they are especially attractive to be incorporated into ion-selective electrodes (ISEs)^18^ for molecular ion detection. ISEs have been studied for over a century beginning with the discovery of pH-sensitive glasses^19^. After decades of work on tuning the glass composition to improve selectivity, ISE research turned to host-guest chemistry and ion-binding receptors (ionophores)^20^,^21^. Incorporating ionophores with ion-exchange sites into a polymer matrix, forming a mixed-matrix membrane (MMM), not only adds mechanical stability from the solid polymeric matrix but also gives tunable permselectivity by setting the ratio of ionophore and ionic sites that are dispersed in an organic liquid matrix^20^. Currently, there are commercially available ionophores and protocols for fabricating potentiometric ISEs with specific binding affinities to about 30 cations and anions^22^. However, most of the ionophores available today are for the detection of elemental and other small ions, despite a great demand for technologies that allow for rapid and precise monitoring of larger organic ions for both biomedical^20^ and environmental^21^,^23^ applications.

Therefore, the present work focuses on the application of MOSCs as new types of size-selective ionophores for the detection of molecular ions. As a demonstrator, MB^+^ (Fig. 1a) was selected as the target ion, since it is a versatile organic molecule widely utilized for a variety of applications in biochemistry and analytical chemistry^24^. For example, MB^+^ has been used as a pH indicator^25^, DNA marker^26^, and gram-negative agar^27^. Although MB^+^ has been proven to be extremely beneficial for laboratory uses, it is also known to have an adverse environmental impact and can be viewed as a water contaminant from textile industries^28^,^29^. Therefore, detecting MB^+^ is both beneficial for monitoring biological systems and valuable for environmental surveillance. In this study, we directly incorporated a MOSC molecule with cavities matching the size of MB^+^ into a polyvinyl chloride (PVC) MMM, and utilized this MMM to fabricate conventional and solid-contact (SC) ISEs. These MOSC based ISEs expressed a near-Nernstian potentiometric response to MB^+^ in the nano- to micro-molar range. Contrast studies with ions of different sizes confirmed the exceptional size-selectivity of the MOSC integrated ISEs. A simulated wastewater experiment was conducted using water from the Fyris River (Sweden), which further proved the practical utility of MOSCs. Moreover, their solution processability, unique multi-cavities adsorption sites, and tunability in structure can enable straightforward designs of a novel ionophore family for rapid and precise monitoring of molecular ions relevant to environmental, biomedical, and other related issues.

## Methods

### Materials

The MOSC, designated as **1-Co**, was synthesized following a published procedure^30^. All purchased chemicals were of analytical or Sigma-Aldrich Selectophore^™^ grade and used as-is, without any further purification. Bis(2-ethylhexyl)sebacate (DOS), high molecular weight poly(vinylchloride) (PVC), potassium tetrakis(4-chlorophenyl) borate (KTpClPB), AgNO_3_, KCl, NH_4_Cl, tetrabutylammonium chloride (TBAC), and ≥99.9% tetrahydrofuran (THF) were purchased from Sigma-Aldrich. Methylene blue was purchased from Merck Millipore. Ag/AgCl pellet (2 mm) was purchased from Warner Instruments. SU8 was purchased from Microchem. Concentration series were prepared using deionized (DI) water (18.2 MΩ·cm) and Fyris River water.

### Mixed-matrix membrane preparation

In brief, 330 mg of PVC and 722 μL of DOS were dissolved in ~5 mL of THF. After the solution became homogeneous, 5.0 mg of KTpClPB and 25.7 mg of **1-Co** were added. The clear solution turned to a transparent light red color after mixing for a few hours. Finally, 3.2 mg of MB^+^ was added and allowed to mix for an additional duration of two hours. The final solution turned from a transparent light red to a dark blue color. A reference MMM solution was fabricated without MOSC inclusion, keeping the rest of the recipe the same.

### Fabrication of conventional and SC-ISEs

Once the solution was mixed, a conventional ISE was fabricated by dipping a disposable pipette tip into the MMM solution, allowing the solution to fill the pipette tip by capillary force. The conventional ISE was completed by inserting a Ag/AgCl pellet into the pipette body and filling the body with a saturated KCl/AgCl solution. Before any measurement was made, the ISEs were conditioned in 10-μM MB^+^ overnight.

The SC-ISE was fabricated by spin-coating a 1.5-μm thick SU8 layer on top of a SiO_2_-covered Si wafer (oxide thickness 650 nm). The SU8 film was then pyrolyzed in a quartz tube flow-through furnace at 900 °C in the reducing atmosphere of 95%N_2_/5%H_2_ for 1 hour. Then, polyethylene wells with inner diameters of ~6 mm were glued on top of the SU8-derived carbon using a quick setting epoxy. The solid-contact ISE was finalized by drop-casting 40 μL of MMM solution inside the fabricated well, on top of the pyrolyzed SU8. After drying for one day, the electrodes were conditioned in 10-μM MB^+^ overnight.

### Measurements

Electrical measurements: The impedance of the MMM was measured at open-circuit potential in the SC-ISE setup using a Bio-Logic VSP-300 potentiostat/galvanostat equipped with an impedance channel and a low-current option. A Ag/AgCl (sat. KCl) reference electrode (RE-1CP, Bio-Logic) and a Pt counter-electrode (Bio-Logic) were used. ISE potentials were measured in reference to the same reference electrode using a Burr-Brown INA116 instrumentation amplifier having an input impedance >1015 Ω. The gain was set at 10. The potential from the preamplifier was digitized using a Keysight U2351A USB data acquisition device at 10 kS/s and smoothed by averaging within a half-second window using National Instruments’ LabView programming environment. In order to minimize any hysteresis during the measurements, the ISE was rinsed thoroughly after each run.

UV-vis measurements: The UV-vis spectra were recorded using a dual-beam PerkinElmer Lambda 950 UV/VIS/NIR Spectrometer. Quartz cuvettes with 10 mm optical length were used.

## Results and Discussion

**Figure 1:**
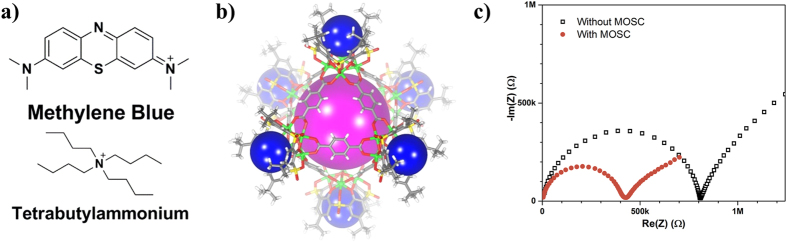
(**a**) Chemical structures of the two molecular cations targeted in this size-selective study: methylene blue (top) and tetrabutylammonium (bottom), (**b**) Structural representation of the MOSC, **1-Co**, studied in this work. The purple and blue spheres indicate the potential binding sites, and (**c**) Impedance measurement of the MMM with and without **1-Co**.

The MOSC chosen for this study, designated as **1-Co** (Fig. 1b), was obtained from the reaction of Co(II), *p*-*tert*-butylsulfonyl-calix[4]arene, and 1,4-benzendicarboxylate^12^. It has an edge-directed octahedral geometry and features an outer diameter of 3.3 nm, an inner diameter of 1.7 nm, and an internal volume of 1.2 nm^3^. It possesses a total of seven well-defined binding domains, including one *endo*- and six *exo*-cavities (Fig. 1b). Recently, we have shown that the **1-Co** MOSC molecule has the ability to selectively bind to MB^+^ in both solution and solid-state^12^,^13^. Specifically, **1-Co** encapsulates ca. 7 equivalents of MB^+^ (i.e., one MB^+^ per cavity) with an apparent binding constant of 1.42 × 10^4^ M.

### Impedance Measurements

The characteristics of the **1-Co** MMM ISEs were investigated using both impedance spectroscopy and potentiometry. The impedance of the MMM with and without **1-Co** is shown in (Fig. 1c). A reduction of the charge-transfer resistance by a factor of two is revealed when comparing the MMM with **1-Co** to the MMM without **1-Co**. The decrease in charge-transfer resistance is a strong indication of **1-Co** promoting MB^+^ ion transfer across the MMM│analyte interface. This was the first sign that the MOSC molecule could be used for potentiometric sensing. It is worth noting that the understanding of the membrane constituents and resulting potential has been well-established^31^. Thus, the concentration-dependence of the phase-boundary potential across the MMM│analyte interface (charge separation layer) is governed by the change in the ratio between free **1-Co** and the complexed **1-Co** within the first few nanometers of the MMM as well as the inclusion of counter-ions (ionic sites)^32^.

### Potentiometric Measurements

**Figure 2:**
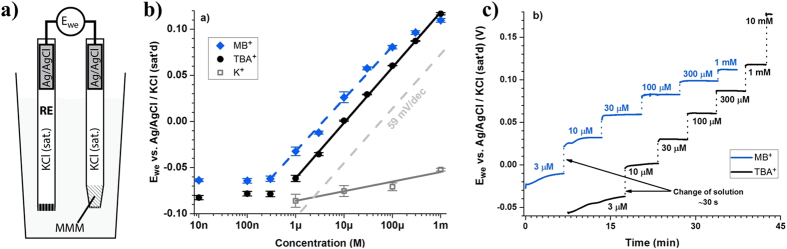
(**a**) Schematic representation of the **1-Co** MMM ISE device, (**b**) Sensitivity of **1-Co** MMM ISE to MB^+^, TBA^+^, and K^+^, and (**c**) response curves of **1-Co** MMM ISE to MB^+^ and TBA^+^.

For the initial potentiometric study, a conventional ISE with an inner filling solution was chosen because it has proven to be extremely versatile and can be easily set up in the laboratory^31^. The potential (E_we_) of the conventional **1-Co** MMM ISE was recorded versus a standard Ag/AgCl reference electrode as depicted in Fig. 2a. The potentiometric results of the **1-Co** MMM ISE shown in Fig. 2b give a near-Nernstian response to [MB^+^] with a slope of 56.8 ± 2.5 mV/p[MB^+^] and a detection limit of ~300 nM. As described above and previously confirmed by single-crystal X-ray diffraction analysis, **1-Co** possesses *endo*- (Ø ~1.7 nm) and *exo*-(Ø ~0.74 nm) cavities that determine its ion-capture properties. The sizes of these cavities fit nicely with the dimensions of MB^+^ that has a length of 1.6 nm and a width of 0.7 nm^33^. Thus, the MB cation can orientate itself to fit into the *endo-*cavity by length and the *exo-*cavity by width. While the binding affinity towards the *endo-* and *exo-* cavities may differ, the effect should not alter the sensitivity as described by the Nernst equation^20^,^21^. The ideal dimensional match of MB^+^ with the MOSC cavities enables the size-selective potentiometric response to MB^+^. This result provides clear evidence for the use of **1-Co** as an ionophore and its direct implementation in ISE sensors.

The **1-Co** MMM ISE was further investigated for its potentiometric response to a concentration series of ions much smaller in size (ionic radii) than MB^+^, including K^+^ (0.275 nm)^34^, Ag^+^ (0.172 nm)^34^, and NH_4_^+^ (0.143 nm)^35^. It is of particular importance to note that the **1-Co** MMM ISE expressed a substantially smaller response to the K^+^ (10.4 ± 2.1 mV/p[K^+^]), Ag^+^ (5.0 ± 7.3 mV/p[Ag^+^]), and NH_4_^+^ (−6.4 ± 1.4 mV/p[NH4^+^]), presumably because their sizes are too small to allow effective binding with the **1-Co** MOSC molecule (Fig. S1). This draws a direct contrast to related calixarene molecules that were found to be efficient in recognizing small metal ions such as Co^2+^, Fe^2+^, Pb^2+^, Ag^+^, etc., through binding sites at their lower rim^36^,^37^,^38^. In comparison, in the case of **1-Co**, the lower rim of its precursor calixarene simply serves as a structural site by coordinating to Co(II) using its phenolic oxygen atoms. While this structural element is fundamental to the formation of the MOSC structure, it diminishes the affinity of the calixarene precursor to small metal ions due to the lack of available binding groups. The lower rim of the sulfonylcalix[4]arene in **1-Co** is further tethered by the 1,4-benzendicarboxylate linker to generate an *endo*-cavity enclosed by aromatic walls (Fig. 1b). This configuration contributes to the selective recognition of bulkier molecular ions by virtue of a size matching and the so-called “cation-π interactions”, a mechanism well recognized in the biological binding of molecular ions (such as neurotransmitters)^39^. In addition, the six *exo*-cavities are each capable of binding to an ionic species that fits within the cavity structures. The highly organized arrangement of these *exo*-cavities around the central *endo*-cavity thus provides an effective strategy to locally concentrate the target ion and thereby enhance the ion recognition as a result. These unique features are not accessible in the calixarene precursor and thus, we conclude that the MOSC-based ionophores are superior for the sensing of molecular ions.

### Determination of size-selectivity

The size selectivity of this particular MOSC ionophore was further investigated by examining the response of the **1-Co** MMM ISE to a cation with a size comparable to MB^+^ but a very different chemical structure. Tetrabutylammonium (TBA^+^; Fig. 1a) was chosen due to its spherical shape with a diameter around 0.8 nm. The near-Nernstian response (60.0 ± 1.5 mV/p[TBA^+^]) of the **1-Co** MMM ISE to TBA^+^ is shown in Fig. 2b. The detection limit was ~1 μM. The response time (for > 90% potential change) of the **1-Co** MMM ISE for MB^+^ and TBA^+^, shown in Fig. 2c, proved to be extremely short, on the order of 1 s (Fig. S2), for concentrations above ~30 μM. The response was slower for lower concentrations with a time duration of 50–100 s, presumably a consequence of slowed ion-exchange kinetics^40^. A good potential stability with a drift that does not exceed a few mV/h is also evident in Fig. 2c. These results confirmed the size selectivity of the MOSC ionophore.

### Incorporation of 1-Co to a solid-contact electrode

To show the versatility of the **1-Co** ionophore, we also fabricated a SC-ISE by applying **1-Co** MMM onto a pyrolyzed SU8-derived carbon (SU8-C) electrode; SU-8 is an epoxy-based photo-patternable polymer. The SC-ISE is considered as a new generation ISE, benefiting from miniaturization, integration into microelectronics, and a decrease in cross-membrane ion fluxes^31^,^41^. E_we_ of the SC-ISE was also recorded versus a Ag/AgCl reference electrode as depicted in Fig. S3a. The **1-Co·**MMM SC-ISE exhibited the same near-Nernstian response to MB^+^ as the conventional setup (Fig. S3b). This result demonstrated the possibility of integrating MOSCs into solid state electronic sensors which can be mass produced with the well-established microelectronics industry at very low costs.

It is noteworthy that once the MMM was cast as either a conventional ISE or a SC-ISE, the stability of both MOSC and MB^+^ in the MMM was exceptional and showed no sign of leaching. A simple leaching test was performed by preparing two MMMs, one with **1-Co** and one without, while keeping all other constituents the same. The MMMs were then stored in deionized (DI) water for over one week. No change in the color of the water for the MOSC MMM was observed since MB^+^ was captured by **1-Co**. However, the solution in contact with the MMM without **1-Co** turned blue, indicating that MB^+^ had leached out (Fig. S4). Notably, both the SC-ISE with a MMM that did not contain **1-Co** and the ISE without MMM altogether (i.e. plain carbon electrode) did not show a pronounced potentiometric response to MB^+^ (Fig. S5). This further confirmed that the MMM ISE sensitivity resulted from the inclusion of the MOSC.

### Proof-of-concept test of 1-Co with river water

**Figure 3:**
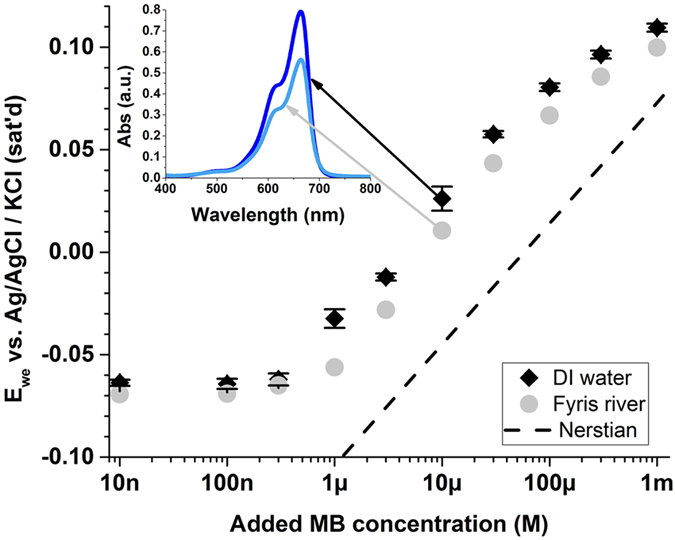
Near-Nerstian response of a simulated wastewater sample (collected from the Fyrisån in Uppsala, Sweden) to MB.

To illustrate that the above proof-of-concept protocols can be applied in a more practical setting that involves a more complex analyte, we conducted a simulated wastewater experiment with water collected from the Fyris River (Fyrisån) in the city of Uppsala. By adding controlled amounts of the “pollutant” MB^+^, the response curve for the simulated wastewater remained near-Nernstian, although an apparent shift of the detection limit was observed (Fig. 3). We attribute this shift to a decrease in the oxygen content of Fyrisån’s water caused by microorganisms found in the river water^42^. The loss of oxygen due to microorganisms after collection of the water sample produces a reducing environment, which can in turn decrease the actual MB^+^ ion concentration by a certain amount and cause a small shift in the response curve. This speculation was confirmed by a control experiment, in which an equal amount of MB^+^ was dissolved in DI water and the water from Fyrisån. The UV-vis absorption showed a lower concentration of MB^+^ in the river water (Fig. 3), indicating that MB^+^ was indeed being partially reduced. It should also be noted that, despite the reduction of MB^+^, the inclusion of wastewater to the system is not expected to change the detection limit of the ISE since the ISE responds to the MB^+^ only in the cationic form. Thus, the detection of MB^+^ by MOSC based ISEs provides a possible method for potentiometric titration of the redox indicator, an exciting aspect we are pursuing in future studies.

## Conclusion

In this study, the **1-Co** MOSC molecule is incorporated into a MMM and subsequently utilized for potentiometric sensing of the molecular ion MB^+^. The unique *endo*- and *exo*-cavities of the MOSC molecule provide locally concentrated and potentially synergistic binding sites for large molecular cations while expressing limited sensitivity towards smaller cations. Our results demonstrate that MOSCs can serve as a new class of size-selective ionophores that are architecturally unique, chemically modular, and functionally robust. The myriad of possibilities to tune the ion recognition capability of the MOSCs by modifying their nanocavity structure, coupled with their ideal compatibility with both conventional and solid-contact ISEs, is anticipated to afford exciting new opportunities in a wide array of sensing applications including environmental monitoring, food quality control, and biomedical surveillance. Many such potential applications remain elusive with currently available technologies.

## Additional Information

**How to cite this article:** Netzer, N. L. *et al*. Biomimetic supercontainers for size-selective electrochemical sensing of molecular ions. *Sci. Rep.* **7**, 45786; doi: 10.1038/srep45786 (2017).

**Publisher's note:** Springer Nature remains neutral with regard to jurisdictional claims in published maps and institutional affiliations.

## Supplementary Material

Supplementary Information
