# Supplementary material for: Biomimetic supercontainers for size-selective electrochemical sensing of molecular ions
Source: Sci Rep. 2017 Apr 10;7:45786. doi: 10.1038/srep45786 (PMC5385547; doi:10.1038/srep45786)
Supplement: Supplementary Information [file srep45786-s1.pdf]

Supplementary information

Biomimetic supercontainers for size-selective electrochemical sensing of molecular ions

Nathan L. Netzer,[1] Indrek Must,[1] Yupu Qiao,[2] Shi-Li Zhang,[1] Zhenqiang Wang,[2,*] and Zhen Zhang[1,*]

1. Solid-State Electronics, The Ångström Laboratory, Uppsala University, SE-751 21, Uppsala, Sweden
2. Department of Chemistry, The University of South Dakota, 414 E. Clark St., Vermillion, SD 57069, United States

*Corresponding authors

Zhen Zhang: zhen.zhang@angstrom.uu.se

Zhenqiang Wang: zhenqiang.wang@usd.edu

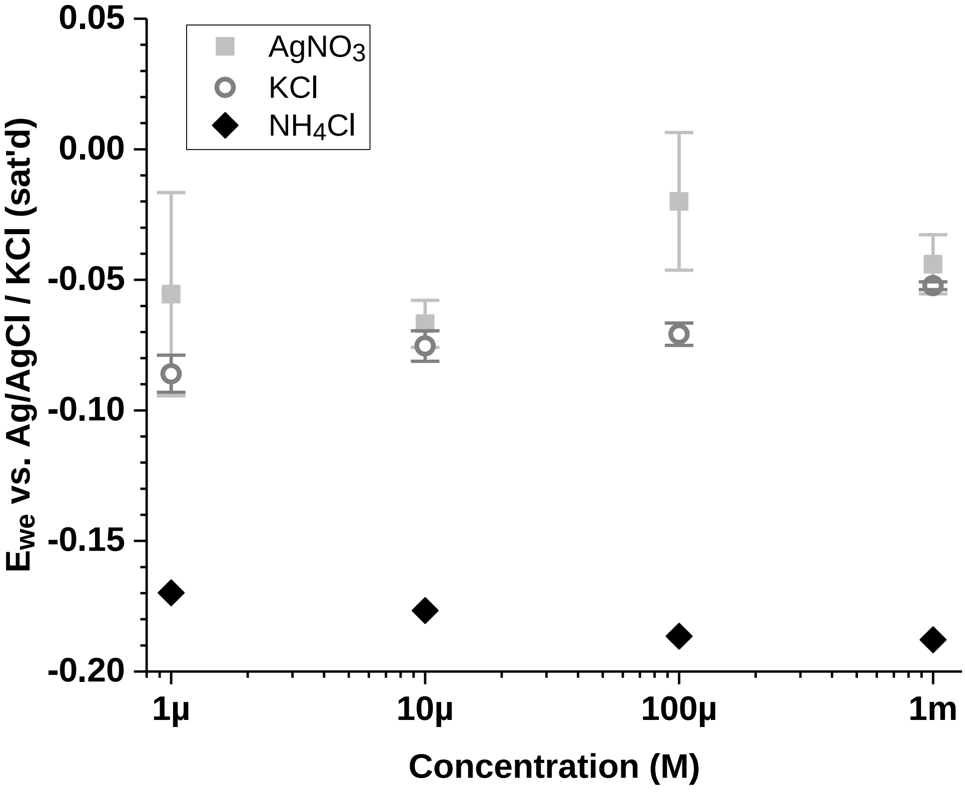

Figure S1. The sensitivity of the **1-Co** MMM ISE for Ag^{+}, K^{+}, and NH_4^{+} cations. In the KCl concentration series, the ionic strength of the electrolytes was not kept constant, while in other measurements, the ionic strength was kept constant using KCl.

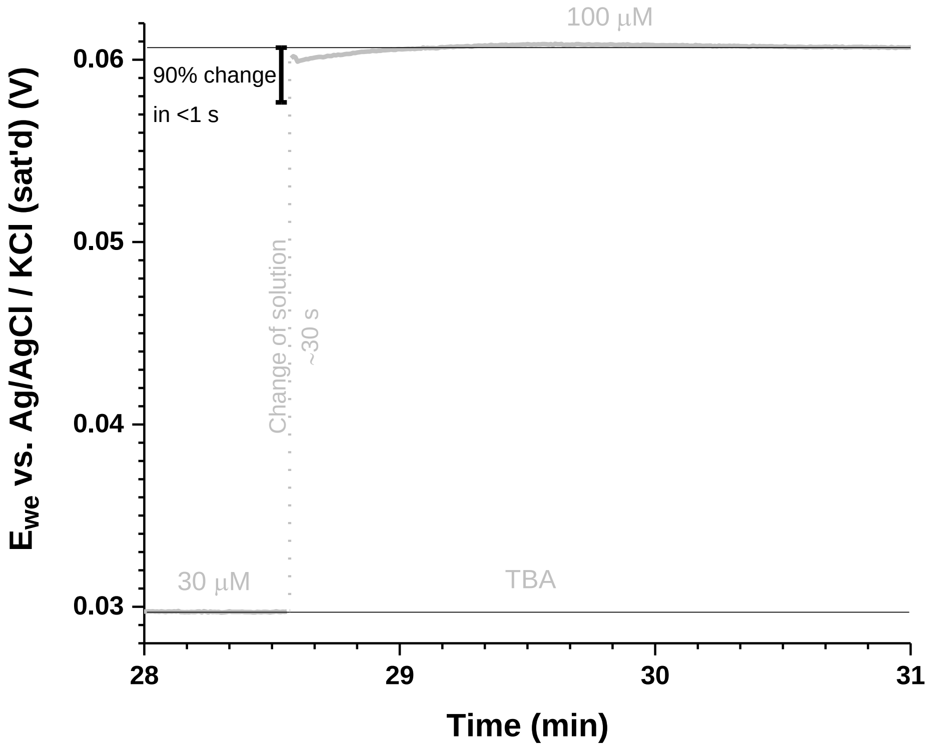

Figure S2. The response time, that is, the time needed to achieve 90% of the equilibrium value, to TBA^+ was well below 1 s in the concentration range of 30–100 μM.

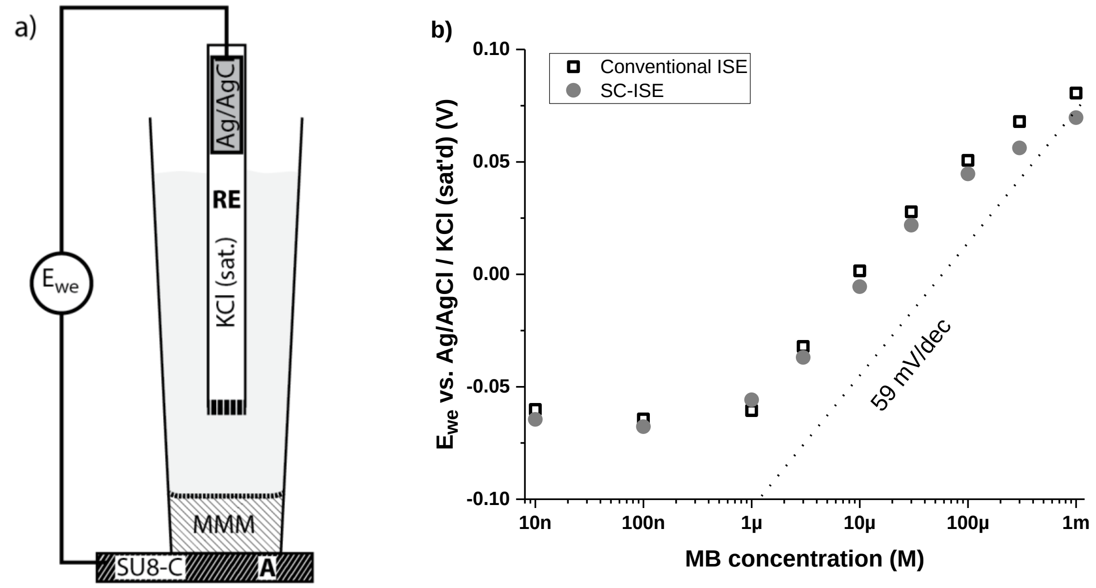

Figure S3. The a) setup for SC-ISE and b) sensitivity comparison of the conventional ISE and SC-ISE to MB^+.

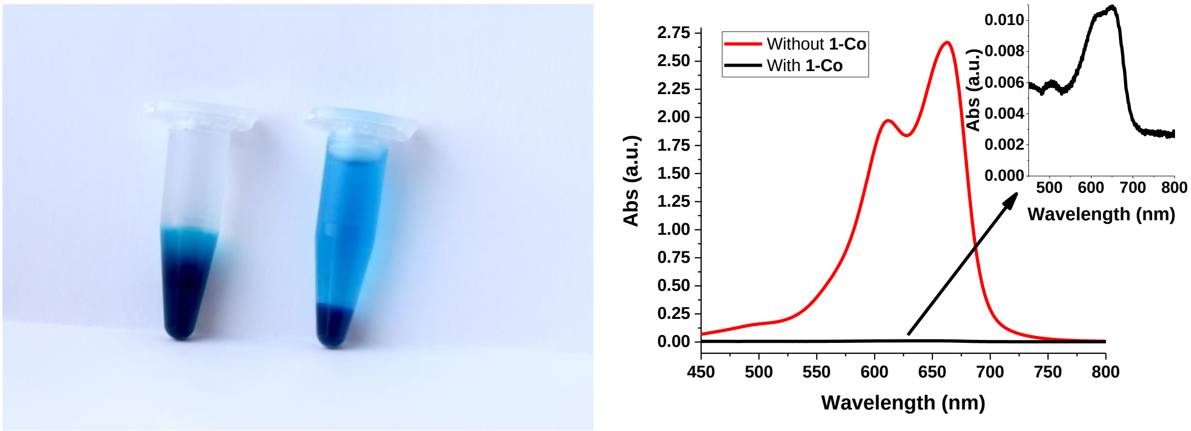

Figure S4. a) Left: In a MMM with both MB and MOSC content, ions are captured strongly and did not significantly leach out into DI water stored over the MMM. Right: MB leached out from a MMM with MB but without MOSC, demonstrating the capturing property of the MOSC. b) The UV-vis spectra corresponding to the aqueous solutions soaking over the two MMMs, demonstrating approximately 270× lower concentration of MB in a solution stored over the MMM with MOSC.

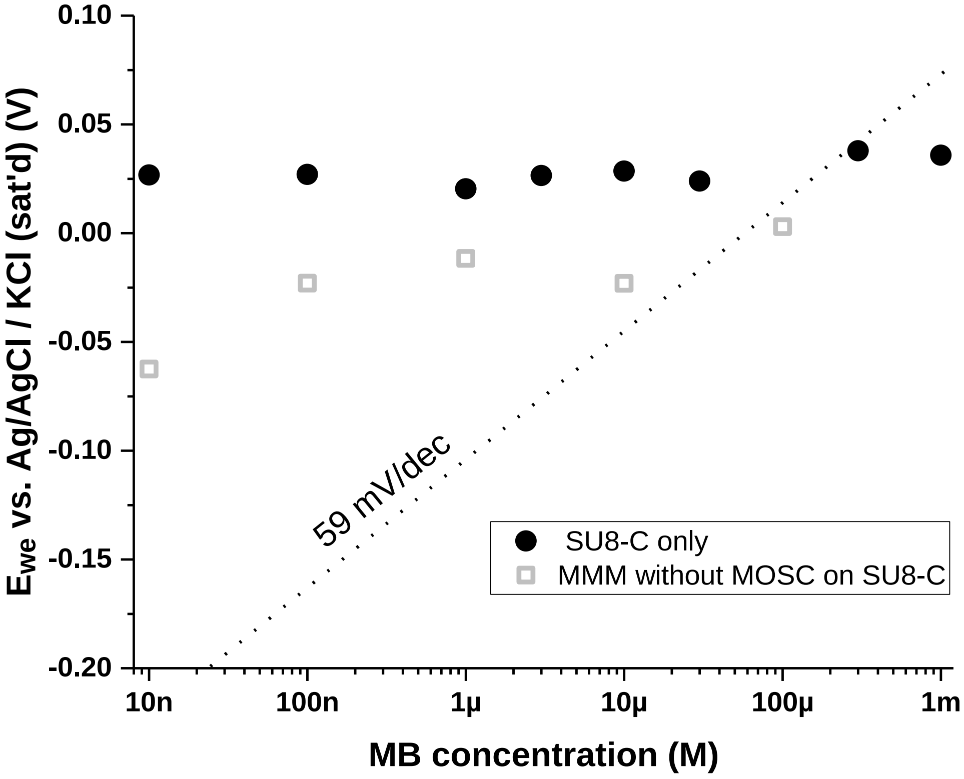

Figure S5. The sensitivity of the two types of SC-ISEs to MB, one without the MMM (carbon only) and the other with the PVC membrane MMM that does not contain MOSC.
